# Study of Protein Haptenation by Amoxicillin Through the Use of a Biotinylated Antibiotic

**DOI:** 10.1371/journal.pone.0090891

**Published:** 2014-03-03

**Authors:** Adriana Ariza, Daniel Collado, Yolanda Vida, María I. Montañez, Ezequiel Pérez-Inestrosa, Miguel Blanca, María José Torres, F. Javier Cañada, Dolores Pérez-Sala

**Affiliations:** 1 Department of Chemical and Physical Biology, Centro de Investigaciones Biológicas, Consejo Superior de Investigaciones Científicas, Madrid, Spain; 2 Research Laboratory Carlos Haya Hospital-IBIMA, Málaga, Spain; 3 Department of Organic Chemistry, University of Málaga, Malaga, Spain; 4 BIONAND-Andalusian Centre for Nanomedicine and Biotechnology, Parque Tecnológico de Andalucía, Málaga, Spain; 5 Allergy Service, Hospital Carlos Haya, Málaga, Spain; Weizmann Institute of Science, Israel

## Abstract

Allergic reactions towards β-lactam antibiotics pose an important clinical problem. The ability of small molecules, such as a β-lactams, to bind covalently to proteins, in a process known as haptenation, is considered necessary for induction of a specific immunological response. Identification of the proteins modified by β-lactams and elucidation of the relevance of this process in allergic reactions requires sensitive tools. Here we describe the preparation and characterization of a biotinylated amoxicillin analog (AX-B) as a tool for the study of protein haptenation by amoxicillin (AX). AX-B, obtained by the inclusion of a biotin moiety at the lateral chain of AX, showed a chemical reactivity identical to AX. Covalent modification of proteins by AX-B was reduced by excess AX and vice versa, suggesting competition for binding to the same targets. From an immunological point of view, AX and AX-B behaved similarly in RAST inhibition studies with sera of patients with non-selective allergy towards β-lactams, whereas, as expected, competition by AX-B was poorer with sera of AX-selective patients, which recognize AX lateral chain. Use of AX-B followed by biotin detection allowed the observation of human serum albumin (HSA) modification by concentrations 100-fold lower that when using AX followed by immunological detection. Incubation of human serum with AX-B led to the haptenation of all of the previously identified major AX targets. In addition, some new targets could be detected. Interestingly, AX-B allowed the detection of intracellular protein adducts, which showed a cell type-specific pattern. This opens the possibility of following the formation and fate of AX-B adducts in cells. Thus, AX-B may constitute a valuable tool for the identification of AX targets with high sensitivity as well as for the elucidation of the mechanisms involved in allergy towards β-lactams.

## Introduction

Protein modification by reactive drugs or their metabolites is an important process in adverse drug reactions. In allergic drug reactions in particular, covalent protein modification by drugs is thought to be necessary to give rise to a structure of sufficient size to trigger an immune response. In this process, the drugs, or haptens, covalently modify proteins (haptenation). Haptenated proteins will be processed by antigen presenting cells and the resulting peptides exposed through MHCI or MHCII-dependent pathways. Alternative mechanisms imply the covalent or non-covalent binding of the drug to the peptides already exposed on the cell surface or to MHC or T-cell receptors [1], [2] (reviewed in [3]). Drug covalent or non-covalent adducts will be engaged by receptors on lymphocytes to elicit a CD4+ or CD8+ cell response or a T-cell response.

β-Lactam antibiotics are the drugs most frequently eliciting allergic reactions. Among the various β-lactams, the trend of allergic reactions has been changing during recent years in correlation with the patterns of prescription and frequency of consumption [4]. Therefore, at present, amoxicillin (AX) is the antibiotic most frequently eliciting allergic reactions [5]. In addition, reactions towards clavulanic acid (CLV) are on the rise [6].

A drawback of diagnostic tests for drug allergy is the fact that the isolated drug or synthetic drug-protein conjugates are often not recognized by patients' drug specific IgE. In addition, antibodies generated against β-lactam conjugates or present in the serum of allergic patients do not recognize equally well the drug when conjugated to different carrier structures [7]–[10]. Similarly, activation of T-cell clones may occur selectively in response to free drug or to drug conjugates [1]. Therefore, accumulating experimental and clinical evidence raises the hypothesis that not only the drug, but parts of the haptenated protein or peptide may contribute important structural determinants for antigen recognition [11]. In this context, identification of haptenated proteins may provide valuable information to understand the mechanisms of allergy as well as to improve the diagnostic procedures.

From a chemical point of view, the reactivity of β-lactam antibiotics depends on the β-lactam ring, which may suffer the attack of various nucleophiles present in proteins, mainly, the amino-terminal groups, the amino groups of the lateral chains of lysine residues, the imidazole ring of histidine residues or the thiol group of cysteine residues [12]. The electrophilic character of the β-lactam ring is related to the strained four member ring next to the thiazolidine ring. The nucleophilic attack results in the opened form of the β-lactam structure, which is stable in the case of penicillins.

From a pathophysiological point of view it has been shown that there is selectivity in the allergic responses and in the recognition of β-lactams by the sera of patients allergic to these antibiotics. Thus, some patients develop allergic reactions selective towards AX but not towards other β-lactams, whereas others suffer allergic reactions towards several β-lactams [5], [11]. Similarly, in diagnostic tests, for some patients binding of IgE present in sera to an immobilized antibiotic can be competed by several β-lactams with similar potency (non-selective allergic patients), whereas for other patients, AX is a more effective competitor (AX-selective allergic patients) [13], [14]. Since the structural feature specific of AX is the lateral chain of the molecule, these observations are interpreted as the antibodies being directed towards this part of the molecule in the “selective” patients and towards the core structure of the molecule in the “non-selective” patients. At present it is not known whether formation of different haptenated structures contributes to this selectivity.

Protein haptenation by β-lactams has been addressed in various studies. Early works on the detection and identification of β-lactam-protein adducts involved HPLC purification, followed by EDMAN degradation [15]. This allowed the identification of several addition sites for penicillin in human serum albumin (HSA), all of which mapped at lysine residues. More recently, both MS and immunological approaches have been used by several groups. Immunological procedures allowed the identification of some serum proteins, including HSA and transferrin, as targets for haptenation by ampicillin [16]. Similarly, an anti-flucloxacillin antibody has been used to detect adducts formed in the liver of flucloxacillin-treated rats [17]. From the various studies available it is inferred that the main target for protein haptenation is HSA. The specificity and sensitivity of the current MS approaches has led to the detailed characterization of the modification of HSA by various β-lactams, both *in vitro* and in samples obtained from patients [12], [18].

Recently, we have used a combination of immunological and high resolution MS approaches to identify several targets for haptenation by AX [12]. Identified targets include HSA, transferrin and heavy and light chains of immunoglobulins. Moreover, we have evidenced that factors independent from protein abundance influence modification by AX. In addition, both, residue reactivity and conformational factors are important for this process. These factors result in the preferential modification of some serum proteins and, in the case of HSA, in the preferential modification of certain residues, particularly Lys190 [12].

Given the importance of the interplay between AX-protein adducts and immune cells for the allergic reaction, we sought to develop approaches to monitor these processes with high sensitivity tools. The avidin-biotin interaction provides great affinity and sensitivity, and the possibility of coupling modification of proteins by biotinylated compounds with several methods for detection, purification and imaging [19], [20]. Here we obtained an AX-B derivative *via* an amide linkage between the amino group of the lateral chain of AX and biotin-NHS ester. In addition, we have explored the potential of AX-B to form adducts with proteins and its suitability as a tool for the study of the mechanisms involved in AX allergy. We show that AX-B is able to form adducts with proteins giving a pattern similar to that given by AX. Moreover, it allows the detection of adducts with 10 to 100-fold higher sensitivity than formerly used immunological methods, allowing the detection of both extra- and intracellular adducts. These findings open new ways for the study of protein haptenation by AX and its importance in the development of allergic reactions.

## Materials and Methods

### Ethics statement

The study was conducted according to the Declaration of Helsinki principles and was approved by the Ethics Committee of Carlos Haya Hospital (Málaga, Spain). All subjects included in the study were informed orally about the study and signed the corresponding informed consent.

### Reagents

Amoxicillin was from Glaxo Smithkline Beecham (Madrid, Spain). Deuterium oxide for NMR studies (D_2_O) was from Panreac Química SAU (Barcelona, Spain). Potassium phosphate buffer (PBS) for ^1^H-NMR reactivity studies was prepared in 99.8% D_2_O at pH 7.4. HRP-Streptavidin and ECL system were from GE Healthcare. Alexa-488 streptavidin was from Molecular Probes. Albumin from human serum, phorbol myristate acetate, iodoacetamide and 4′,6-diamidino-2-phenylindole dihydrochloride (DAPI) were from Sigma. Biotin-NHS ester was from Aldrich.

### Preparation of AX-B

Amoxicillin (77 mg, 0.2 mmol) and Biotin-NHS ester (68 mg, 0.2 mmol) were placed in a 10 ml round bottom flask. The flask was degassed. Once the argon atmosphere was achieved, the flask was introduced in an ice-water bath (0°C) and 3 ml of methanol was added. The mixture was stirred for 5 minutes and then 35 μl (0.2 mmol) of triethylamine was added. The reaction was stirred for 2 hours in the ice-water bath, ensuring that the temperature was kept at 0°C, and 1 hour at room temperature (25–27°C).

The reaction mixture was lyophilized and a white solid obtained, corresponding to the AX-B triethyl ammonium salt.

Yield 80%. White solid. **^1^H-NMR** (400 MHz, D_2_O) δ ppm: 7.36 (d, 2H, *J* = 8.7 Hz, Ar), 6.97 (d, 2H, *J* = 8.7 Hz, Ar), 5.55–5.48 (m, 2H, H_5_, H_6_), 5.46 (s, 1H, H_10_), 4.66–4.61 (m, 1H, H_20_), 4.40–4.37 (m, 1H, H_24_), 4.23 (s, 1H, H_3_), 3.33–3.28 (m, 1H, H_17_), 3.25 (q, 6H, *J* = 7.3 Hz −C*H_2_*CH_3_), 3.07–3.00 (m, 2H, H_19_), 2.84–2.80 (m, 2H, H_16_), 2.39 (t, 2H, *J* = 7.1 Hz, H_13_), 1.79–1.66 (m, 4H, H_14_, H_15_), 1.53 (s, 3H, −CH_3_), 1.49 (s, 3H, −CH_3_), 1.33 (t, 9H, *J* = 7.3 Hz, −CH_2_C*H_3_*). EI-MS *m/z* (%): 86 (100), 100 (48), 107 (27), 166 (44), 183 (23), 185 (7), 227 (5), 335 (1).

### Evaluation of AX-B reactivity

A solution of AX or AX-B with butylamine in deuterated solvent was added to appropriate NMR tubes, as described the following: each amoxicillin (15·10^−6^ mol) was dissolved in 0.5 ml of deuterated solvent (D_2_O or deuterated PBS), and an excess of butylamine (5·10^−6^ l, 50·10^−6^ mols) was added. The reactions were monitored by ^1^H NMR on a Bruker ASCENDTM 400 MHz.

### *In vitro* formation and detection of AX-HSA and AX-B-HSA adducts

HSA was dissolved at 10 mg/ml in PBS pH 7.4 or 50 mM Na_2_CO_3_/NaHCO_3_ pH 10.2, as indicated, and incubated with various concentrations of AX or AX-B at 37°C for 16 h. Before analysis, the incubation mixture was diluted in PBS and aliquots containing 2 μg of protein were loaded onto 10% polyacrylamide gels. After SDS-PAGE, gels were transferred to Immobilon P membranes using a semi-dry transfer system from Bio-Rad. For immunological detection, blots were incubated with AO3.2 anti-AX adducts monoclonal antibody [21] at 1∶1000 dilution, followed by secondary antibody HRP-conjugated anti-mouse immunoglobulins (Igs) at 1∶2000 dilution, and proteins of interest were detected with ECL. For detection of AX-B-HSA adducts, blots were incubated with HRP-streptavidin (1∶1000 dilution), and biotin-positive signals were detected with ECL.

### *In vitro* modification and analysis of serum proteins modified by AX or AX-B

Human serum obtained from healthy donors (Sigma) was incubated at 30 mg of protein/ml in the absence or presence of AX or AX-B, as indicated, in PBS at 37°C for 16 h. For SDS-PAGE analysis, serum samples were diluted with PBS and aliquots containing 2 μg of protein were loaded onto 12.5% polyacrylamide gels. For 2D analysis, 70 μg of protein were precipitated with chloroform:methanol, using the method of Wessel and Flugge [22]. The precipitated proteins were resuspended in 140 μl of IEF sample buffer (4% CHAPS, 2 M thiourea, 7 M urea, 100 mM DTT, and 0.4% Bio-lyte ampholytes) and loaded on ReadyStrip IPG Strips (pH 3–10, Bio-Rad) for isoelectric focusing on a Protean IEF cell (Bio-Rad), following the instructions of the manufacturer. Before the second dimension, strips were equilibrated in 6 M urea, 2% SDS, 0.375 M Tris, pH 8.8, 20% (vol/vol) glycerol, containing 130 mM DTT for the first equilibration step and 135 mM iodoacetamide for the second step. Strips were then placed on top of 12.5% polyacrylamide SDS gels. Detection of modified proteins was performed as above.

### Cell culture and treatments

RAW264.7 murine macrophage cells from the American Tissue Culture Collection (ATCC, Manassas, VA) were cultured in DMEM plus 10% (vol/vol) fetal bovine serum (FBS), 100 U/ml penicillin and 100 μg/ml streptomycin in a humidified atmosphere, as previously described [23]. THP-1 human acute monocytic leukemia cells (ATCC) and RPMI 8866 human B-lymphocyte cells [24] were cultured in RPMI 1640 supplemented with serum and antibiotics. When indicated, cells were cultured in antibiotic-free medium 48 h before treatment with AX or AX-B. After treatments, cells were washed with PBS and lysed in 50 mM Tris-HCl, pH 7.5, 0.1 mM EDTA, 0.1 mM EGTA, 0.1 mM 2-mercaptoethanol, 1% NP-40 or 0.5% SDS, containing 0.1 mM sodium orthovanadate, 50 mM sodium fluoride and protease inhibitors (2 μg/ml each of leupeptin, aprotinin and trypsin inhibitor, and 0.32 mg/ml AEBSF).

### Confocal microscopy

RAW264.7 cells grown on glass coverslips were cultured in the absence or presence of AX-B at 0.5 mg/ml for 24 h. For visualization of biotin-positive structures cells were fixed with 4% paraformaldehyde for 30 min and stained with Alexa488-streptavidin, as previously described [19]. Nuclei were counterstained with DAPI. Fluorescence images shown are single z sections obtained with a Leica DMRE2 confocal microscope.

### Patients

The study of the reactivity of sera from allergic patients towards AX-B included patients who had been diagnosed with an immediate allergic reaction to AX using the diagnostic procedure described in the ENDA protocol [25]. Patients were classified into two groups: Group A (patients with allergic reactions selective to AX), those with a skin test or drug provocation test (DPT) positive to AX and confirmed good tolerance to benzylpenicillin (BP) by DPT; Group B (patients with allergic reactions cross-reactive to BP and AX), those with positive skin test to BP determinants or positive DPT to BP. Subjects with negative skin test to both BP and AX determinants and with good tolerance to AX were used as controls.

### Skin testing

Skin testing was carried out as described [25], using 0.03 ml of solution prepared daily. The reagents used were penicilloyl polylysine (PPL) (Diater, Madrid, Spain) at 5×10^−5^ and 5×10^−4^ mM, minor determinant mixture (MDM) (Diater, Madrid, Spain) at 2×10^−2^ and 2×10 mM, and AX (Glaxo Smithkline, Madrid, Spain) at 2 and 20 mg/ml.

In the skin prick tests, a wheal larger than 3 mm with a negative response to the control saline was considered positive. In the intradermal tests, the wheal area was marked initially and 20 min after testing, and an increase in diameter greater than 3 mm was considered positive [26].

### Drug provocation test

Drug provocation test (DPT) was carried out in all patients in a single-blind procedure following the previously described protocol [25], [27] in accordance with the ENDA recommendations. DPT consists in the administration of increasing doses of BP (Normon Laboratories, Madrid, Spain) and/or AX (Glaxo Smithkline, Madrid, Spain) at regular intervals of time (30–60 minutes) to achieve the therapeutic dose. The test was considered positive when symptoms compatible with an immediate response appeared with any of the incremental concentrations used.

### Specific IgE antibodies determination

Levels of serum specific IgE from patients were quantified by using radioallergoabsorbent test (RAST) as previously described [28]. BP (Normon Laboratories, Madrid, Spain) and AX (Glaxo Smithkline, Madrid, Spain) at 20 mg/ml were conjugated to poly-L-lysine (PLL) (Sigma, St Louis, MO, USA) at 10 mg/ml previously bound to cyanogen bromide-activated cellulose discs (n° 54, Whatman International Ltd, Maidstone, Kent, United Kingdom) as described [7], [29] and control discs with PLL and no conjugated drugs were also obtained. RAST was performed following the protocol described by Wide et al. [30] by incubating sera from patients with the solid phase for 3 hours. Radiolabelled anti-IgE antibody with I^125^ (kindly provided by ALK-Abello, Madrid, Spain) was then added and samples were incubated overnight. The radioactivity was then measured in a gamma counter (Packard BioScience Company, Frankfurt, Germany) as counts per minute (cpm). Results were calculated as a percentage of the maximum of cpm (% RAST) and considered positive if they were higher than 2.5% of label uptake, which was the mean ±2 SD of the negative control group.
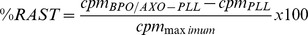



### Determination of IgE specificity and cross-reactivity: RAST inhibition

RAST inhibition was performed as previously reported [7], [28], [31]. Sera from patients with RAST values higher than 7% to both benzyl penicilloyl-PLL (BPO-PLL) and amoxicilloyl acid-PLL (AXO-PLL) or only to AXO-PLL were incubated with AX or AX-biotin at 10-fold serial dilutions (100, 10 and 1 mM, final concentrations) in PBS for 3 hours. Then, solid phase conjugated with AXO-PLL were added and the RAST procedure continued as described above. Results were expressed as percentage of inhibition calculated according to the following formula:
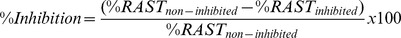



## Results

### Synthesis and characterization of AX-B

**Figure 1:**
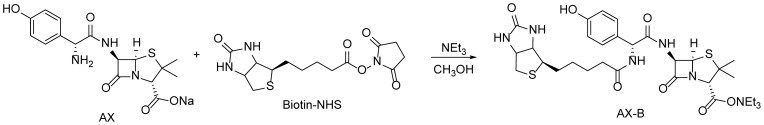
Synthetic procedure for AX-B.

**Figure 2:**
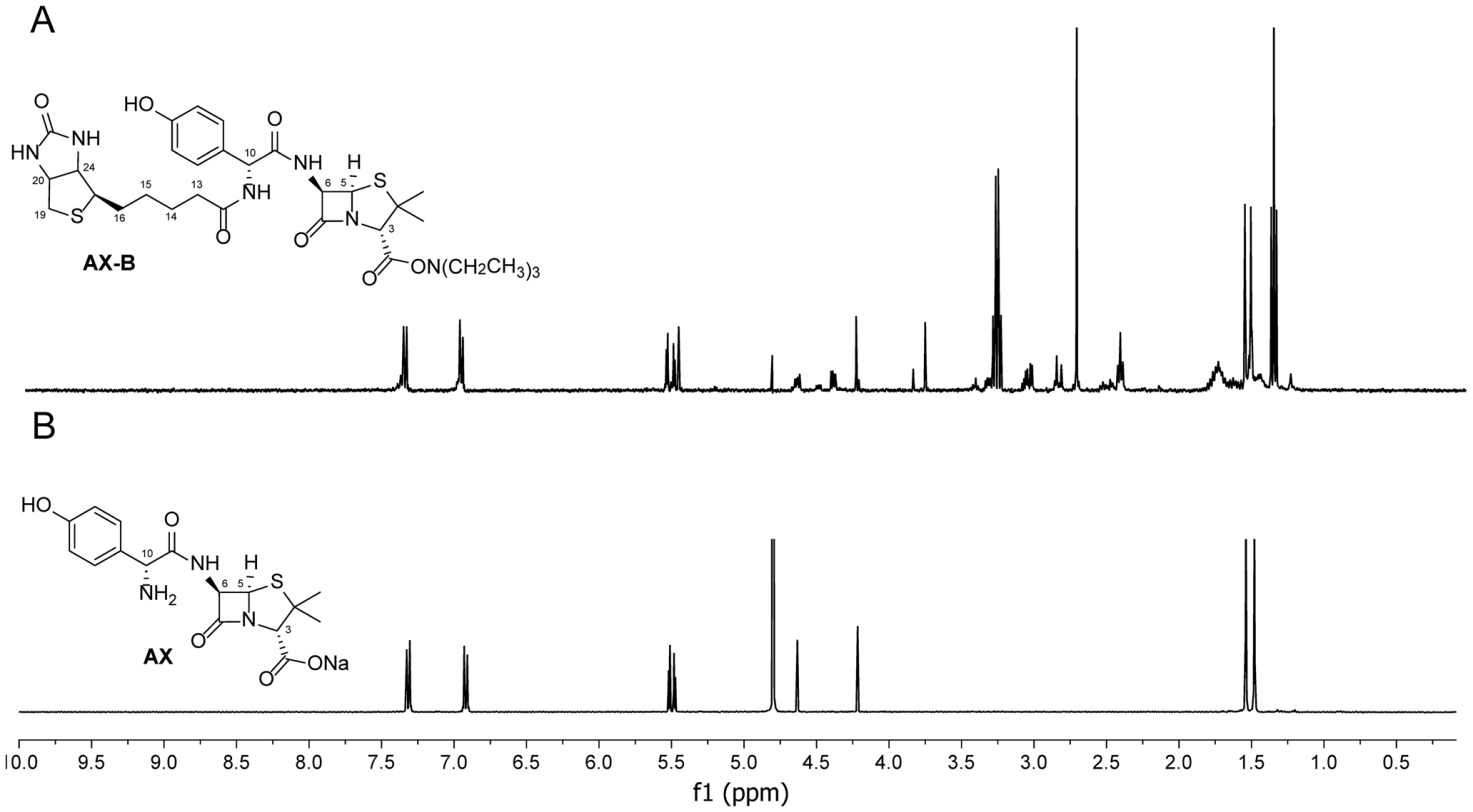
^1^H-NMR spectra of (A) AX-B and (B) AX recorded in D_2_O.

The derivatization of AX with biotin NHS-ester was carried out as described in the Experimental Section. The reaction was optimized until there were no evidences of β-lactam ring opening. A schematic view of the reaction and the resulting compound (AX-B) is shown in Fig. 1. ^1^H-NMR analysis of AX-B confirms the chemical structure of the AX-B derivative (Fig. 2).

### AX and AX-B display similar reactivity towards butylamine

**Figure 3:**
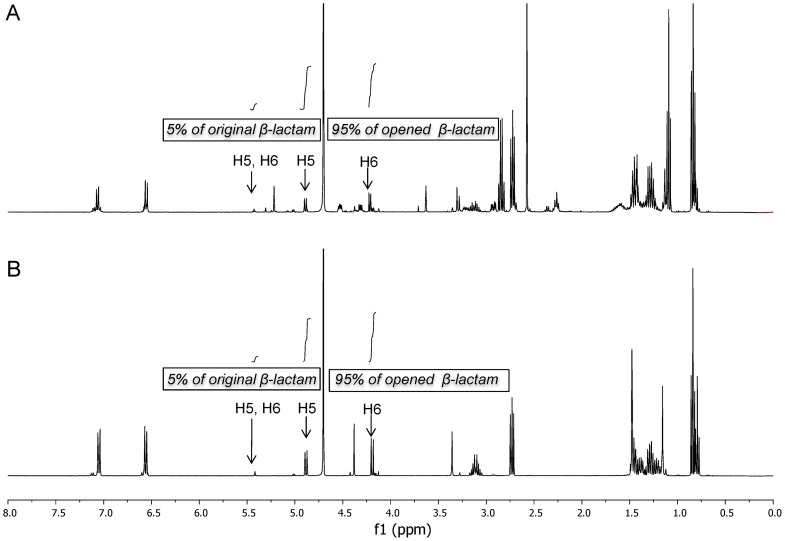
Reactivity of AX and AX-B. ^1^H NMR of each amoxicillin: (A) AX-B and (B) AX, in presence of butylamine in D_2_O after 15 min. Arrows point at signals corresponding to H5 and H6, used to monitor the reaction.

We compared the acylation abilities of AX and AX-B towards a simple nitrogen nucleophile by reaction of either β-lactam with butylamine at NMR scale. Butylamine mimics the lateral chain of lysine, and conjugates of butylamine to various penicillins have been previously used for epitope mapping of β-lactam antibiotics [21]. The characteristic ^1^H chemical shifts for protons H-5 and H-6 in the β-lactam ring of AX were used to monitor the reaction. First, we quickly tested the reactivity using D_2_O as solvent, and after 15 minutes, both β-lactams yielded 95% of amide linkage with butylamine (Fig. 3). In addition, these reactions were carried out in similar conditions to our protein experimental studies (pH 7.4, 37°C and 16 h of incubation time), and only the products corresponding to the aminolysis of the β-lactams were detected (data not shown).

### Modification of HSA by AX and AX-B

**Figure 4:**
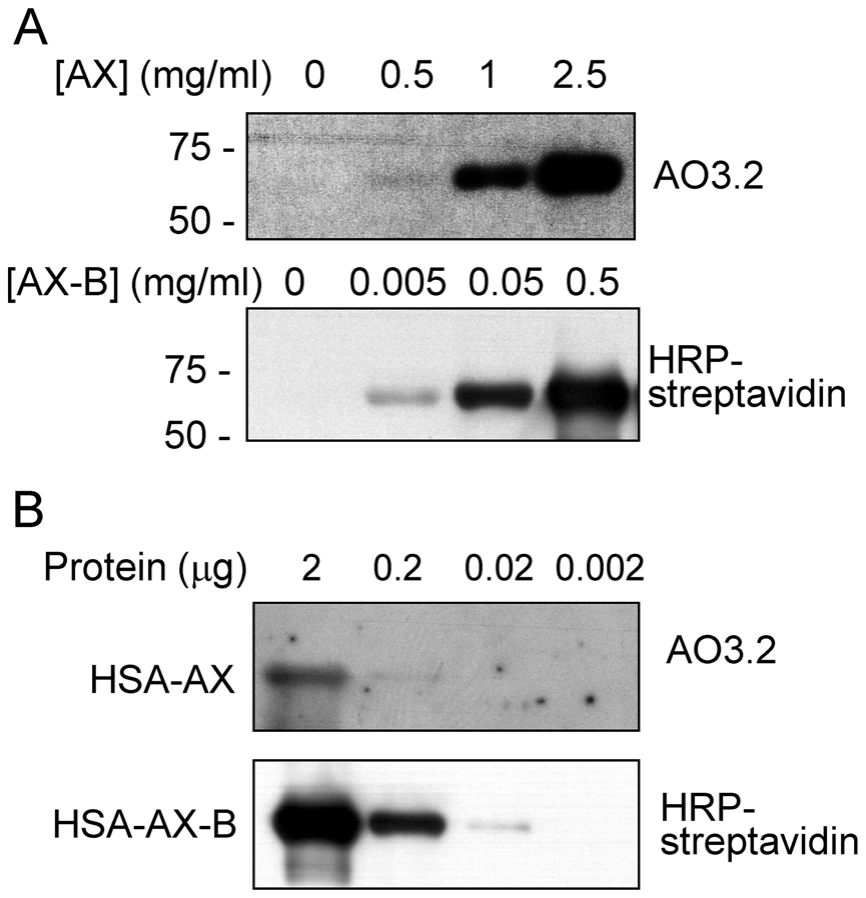
Modification of HSA by AX and AX-B. (A) HSA was incubated in the presence of the indicated concentrations of Ax or Ax-B and adduct formation was assessed by western blot and detection with an anti-AX antibody (AO3.2) or with HRP-streptavidin. Exposure times were 5 minutes for AX and one second for AX-B detection, respectively. (B) HSA was incubated with AX or AX-B at 0.5 mg/ml for 16 h at 37°C. Aliquots of the incubation mixture containing the indicated amounts of total protein were analyzed by SDS-PAGE and adducts formed were detected as above. Exposure times were 2 minutes for AX and one second for AX-B detection, respectively.

HSA is one of the major targets for haptenation by AX in human serum. As we previously described [12], the modification of HSA by AX could be detected by Western blot using the anti-AX monoclonal antibody AO3.2 (Fig. 4). Under the conditions of our assay, this procedure allowed the detection of HSA modified in the presence of 0.5 mg/ml AX at neutral pH (Fig. 4A). Remarkably, in the case of AX-B, the HSA adduct could be detected after incubation of the protein with concentrations of the antibiotic as low as 5 μg/ml. The superior sensitivity of Ax-B was also evidenced by a dilution approach (Fig. 4B). HSA was modified by incubation of AX or AX-B at 0.5 mg/ml and decreasing amounts of the incubation mixtures were analyzed by SDS-PAGE and blot followed by immunological or HRP-streptavidin detection. Using this approach, detection of AX-modified HSA required at least 0.2 μg of protein and long exposure times, whereas modification by AX-B could be clearly detected with 0.02 μg after 1 s exposure, whereas after 1 min, modification could be detected even with 0.002 μg of protein (not shown).

**Figure 5:**
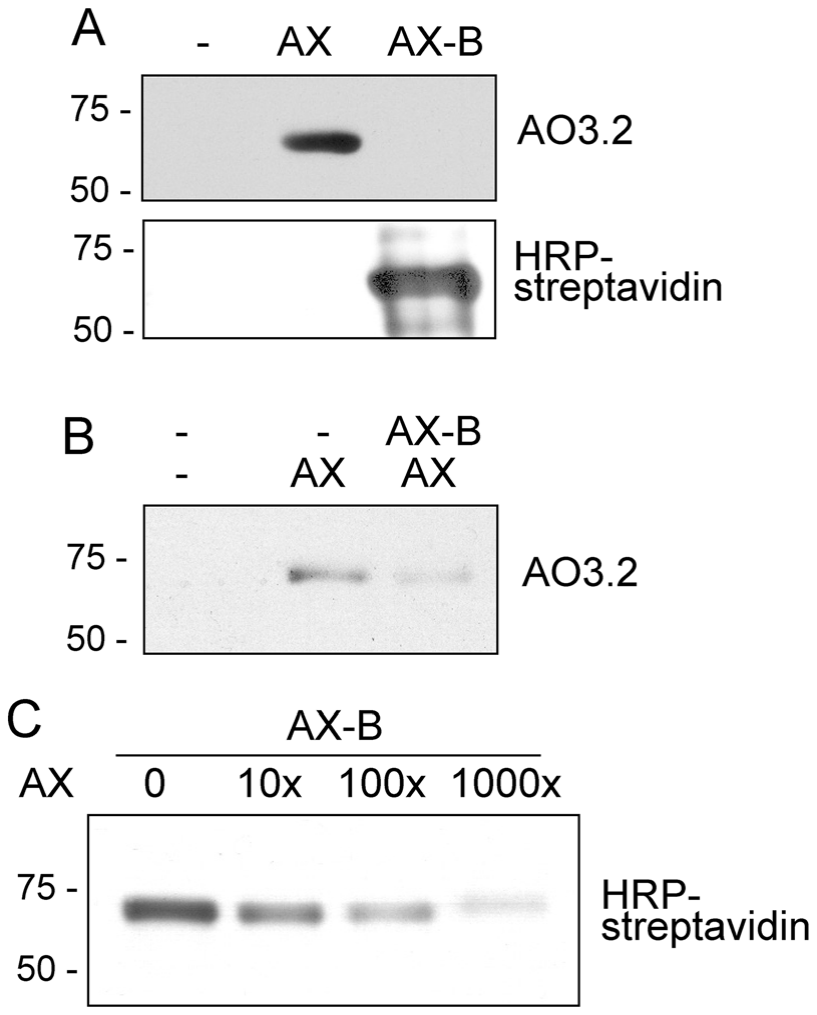
Competition between AX and AX-B for modification of HSA. (A) HSA was incubated overnight with a 9-fold molar excess of AX or AX-B in bicarbonate buffer and detection of adducts was achieved by western blot with an anti-AX antibody (AO3.2) or with HRP-streptavidin, as indicated. (B) HSA was incubated in the absence or presence of AX-B, as above, followed by an overnight incubation with AX. Binding of AX was assessed by western blot with AO3.2 antibody. (C) HSA was incubated for 2 h with 80 μM AX-B, after a 16 h pre-incubation with the indicated concentrations of AX, expressed in molar excess with respect to AX-B. Aliquots of the incubation containing 2 μg of protein were subjected to SDS-PAGE and transfer, and incorporation of AX-B was assessed by detection with HRP-streptavidin. Blots shown in every case are representative of three independent assays with similar results.

The monoclonal antibody AO3.2 recognizes the lateral chain of AX [21]. Consistent with this specificity, this antibody did not recognize HSA modified by AX-B, since in this compound the lateral chain is altered by the introduction of the biotin moiety (Fig. 5A). Therefore, we took advantage of this specificity to explore whether there was a competition between both antibiotics for binding to HSA. Incubation of HSA in the presence of an excess of AX-B precluded the incorporation of AX, as detected with the AO3.2 antibody (Fig. 5B). Conversely, pre-incubation of HSA with an excess of AX reduced the incorporation of AX-B, as evidenced by incubation with HRP-streptavidin and ECL detection (Fig. 5C).

### Recognition of AX-B by the serum of patients allergic to AX

Next, we wanted to assess the ability of sera from allergic patients to recognize AX-B. Thus, the validation study included 8 patients (5 men and 3 women) with immediate allergic reactions to AX (Table 1). 4 subjects were included in group A (showing selectivity towards AX) and 4 subjects in group B (showing cross-reactivity with BP).

**Table 1:** Clinical characteristics and skin test and drug provocation test results of the patients included in the study.

Patient	Group	Age (y)	Sex	Time interval reaction-study (months)	Reaction	Drug	Time interval drug-reaction (min)	Skin test			DPT	
								PPL	MDM	AX	BP	AX
1	A	43	M	8	anaphylaxis	AX-CLV	80	−	−	+	−	nd
2	A	54	F	8	Anaphylactic shock	AX	Less than 60	−	−	+	−	nd
3	A	43	M	4	anaphylaxis	AX-CLV	Less than 60	−	−	+	−	nd
4	A	55	M	2	Urticaria angioedema	AX-CLV	10	−	−	+	−	nd
5	B	18	F	6	anaphylaxis	BP	5	+	−	+	nd	nd
6	B	36	M	0.6	Anaphylactic shock	AX	10	+	−	+	nd	nd
7	B	61	F	2	Urticaria	AX-CLV	120	+	+	+	nd	nd
8	B	51	M	6	anaphylaxis	AX	120	+	−	+	nd	nd

y: years; PPL: penicilloyl-polylysine; MDM: minor determinant mixture; AX: amoxicillin; CLV: clavulanic acid; DPT: drug provocation test; BP: benzylpenicilloyl; nd, not done.

**Figure 6:**
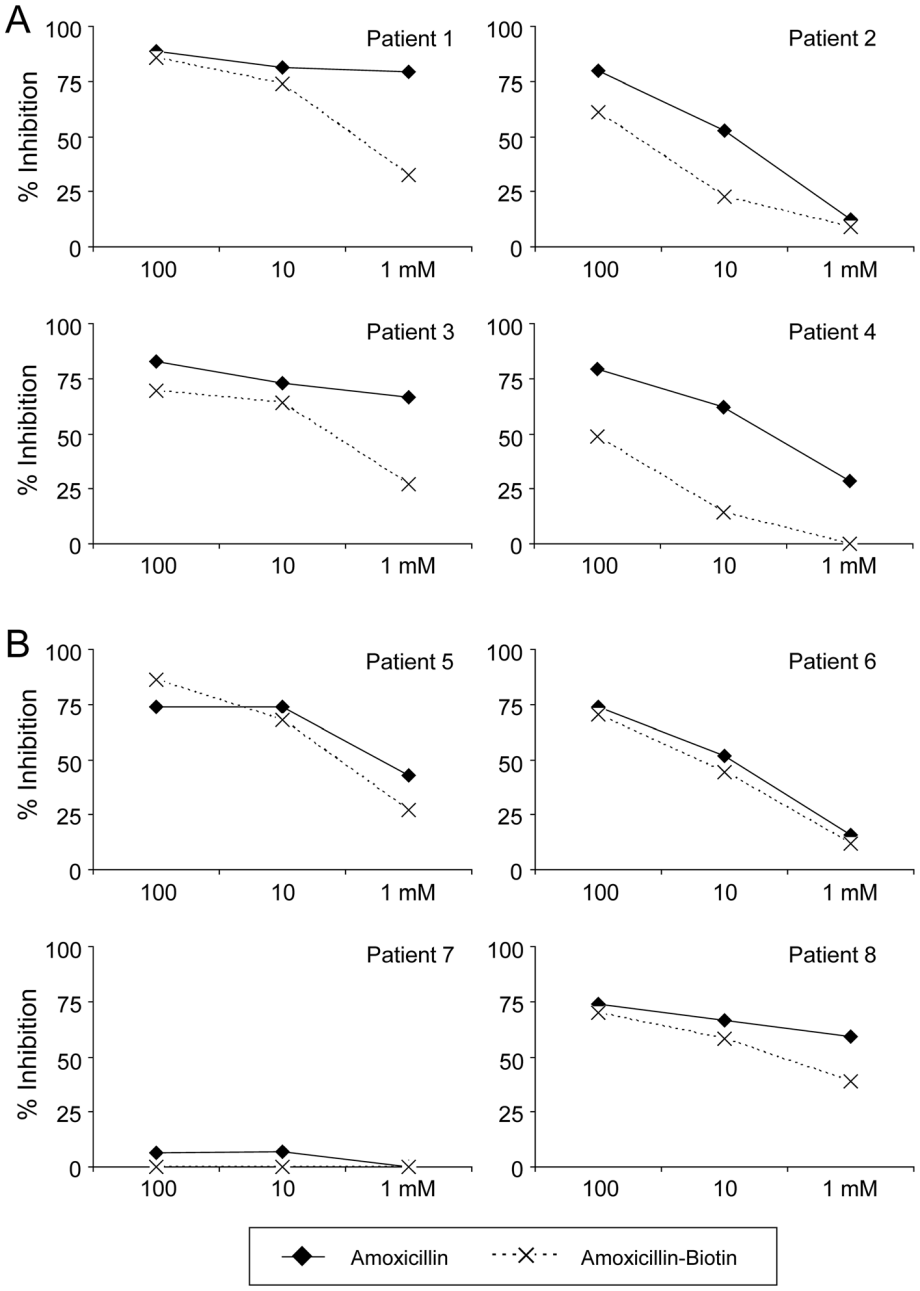
Recognition of AX and AX-B by the sera of allergic patients. Shown are RAST inhibition assays using in the solid phase AXO-PLL discs and in the fluid phase AX and AX-biotin, at the indicated concentrations. Results are from selective allergic patients to AX (A, patients 1–4) and from cross-reactive allergic patients (B, patients 5–8).

RAST inhibition studies were performed in 8 cases with a high RAST binding value (>7%), 4 patients from group A (1A, 2A, 3A and 4A) and 4 patients from group B (5B, 6B, 6B and 8B). We observed a better recognition of AX than AX-B in assays using serum from patients with IgE specific for AX (group A, AX-selective patients), which recognizes mainly the lateral chain structure of AX (Figure 6A, Table 2). Nevertheless, specific serum IgE antibodies from cross-reactive patients (group B), which recognize the structure of the molecule common to other penicillins, namely, the 6-aminopenicillanic acid, recognized both AX and AX-B with similar affinities (Figure 6B, Table 2). Results obtained in these assays suggested that the conjugation of biotin to the side chain amino group of AX interferes with the recognition of this structure in the case of selective allergic reactions. No significant inhibition was obtained in the assays made with control sera, which suggested that biotin conjugated to AX is not non-specifically recognized by total serum IgE antibodies (data not shown).

**Table 2:** RAST and RAST inhibition results from patients with RAST values higher than 7% to AXO-PLL and/or BPO-PLL.

Patient	% RAST		Inhibitor	% RAST inhibition		
	BPO-PLL	AXO-PLL		100 mM	10 mM	1 mM
1	0.00	25.58	AX	88.5	81.3	79.1
			AX-biotin	85.6	74.1	32.4
2	0.20	33.11	AX	79.6	52.9	12.4
			AX-biotin	61.3	22.6	9.0
3	1.68	29.68	AX	82.6	73.1	66.7
			AX-biotin	69.5	64.1	26.9
4	0.00	29.80	AX	79.2	62	28.5
			AX-biotin	48.6	14.4	0.0
5	51.17	26.30	AX	74.0	74.0	42.7
			AX-biotin	86.0	68.0	27.3
6	25.53	28.92	AX	74.0	51.7	15.9
			AX-biotin	70.2	44.1	11.7
7	39.96	42.82	AX	6.6	7.1	0.0
			AX-biotin	0.0	0.0	0.0
8	28.61	33.28	AX	74.1	66.7	59.3
			AX-biotin	69.8	58.0	38.9

### Modification of serum proteins by AX and AX-B

**Figure 7:**
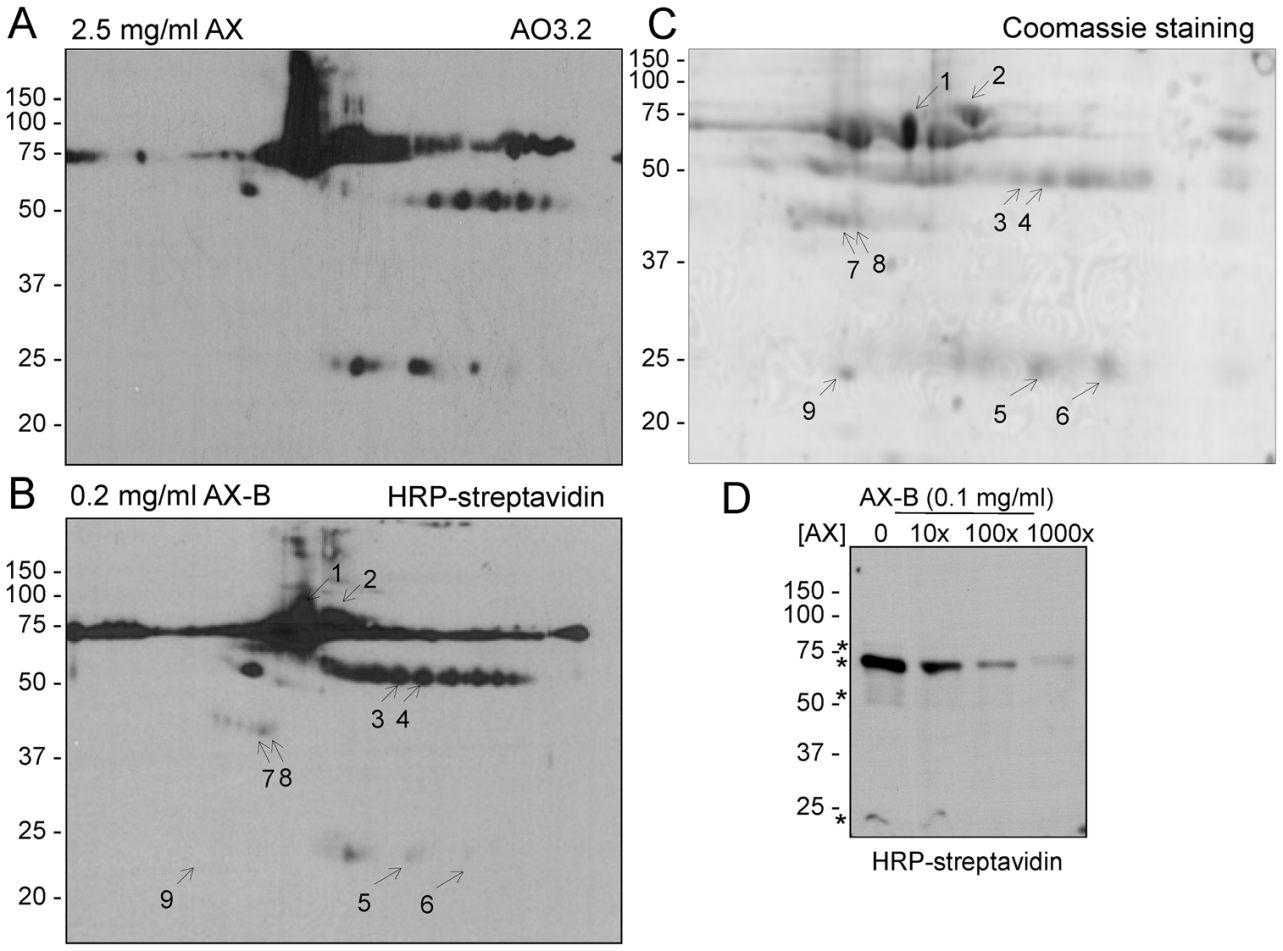
Modification of serum proteins by AX-B. Total human serum was incubated in the presence of the indicated concentrations of AX or AX-B, for 16 h, after which, 70 μg of protein were analyzed by 2D electrophoresis, as described in the experimental section. (A) and (B) AX and AX-B-modified proteins were visualized by detection with AO3.2 antibody or HRP-streptavidin, as indicated. Exposure times were 3 min and 5 seconds, respectively. (C) Coomassie staining of one gel showing the positions of the identified proteins. 1, HSA; 2, transferrin; 3, 4 heavy chains of Igs; 5, 6, light chains of Igs; 7, 8, haptoglobin 2; 9, apolipoprotein A1. (D) Total human serum was incubated with 0.1 mg/ml AX-B (0.16 mM) for 2 h after a 16 h pre-incubation with the indicated concentrations of AX, expressed in molar excess with respect to AX-B. Aliquots of the incubations containing 0.5 μg of protein were resolved on a 10% polyacrylamide gel, before transfer and detection with HRP-streptavidin. The positions of transferrin, HSA and heavy and light chains of Igs, from top to bottom, are marked by asterisks.

As previously described by us and shown in Fig. 7A and C, besides HSA, other serum proteins may be targets for AX modification [12]. Therefore, we next explored the ability of AX-B to form adducts with serum proteins. As shown in Fig. 7B, incubation of human serum with AX-B led to the formation of adducts with various proteins, which could be detected by HRP-streptavidin after 2D-electrophoresis. Most of biotin-positive spots coincided with those modified by AX and detected by western blot with AO3.2, and corresponded to those previously identified as HSA (spot 1), light and heavy chains of immunoglobulins (spots 3,4 and 5,6, respectively) and transferrin (spot 2) [12]. The position of the targets in the Coomassie-stained membrane is shown in Fig. 7C. Moreover, as observed with purified HSA, AX-B allowed the detection of protein adducts with higher sensitivity, with at least 10-times lower concentrations of AX-B being required to elicit a detectable modification. This allowed the observation of a biotin signal associated with haptoglobin 2 (spots 7 and 8), which was negative with AO3.2. Apolipoprotein A1 (spot 9) was negative with both procedures. For detailed MS data on the identification of the various serum proteins please see [12]. Control samples gave negligible background after either immunological or biotin detection. Similar to above, incubation of human serum in the presence of an excess AX reduced the incorporation of AX-B into serum proteins (Fig. 7D).

### Detection of intracellular protein adducts with AX-B

**Figure 8:**
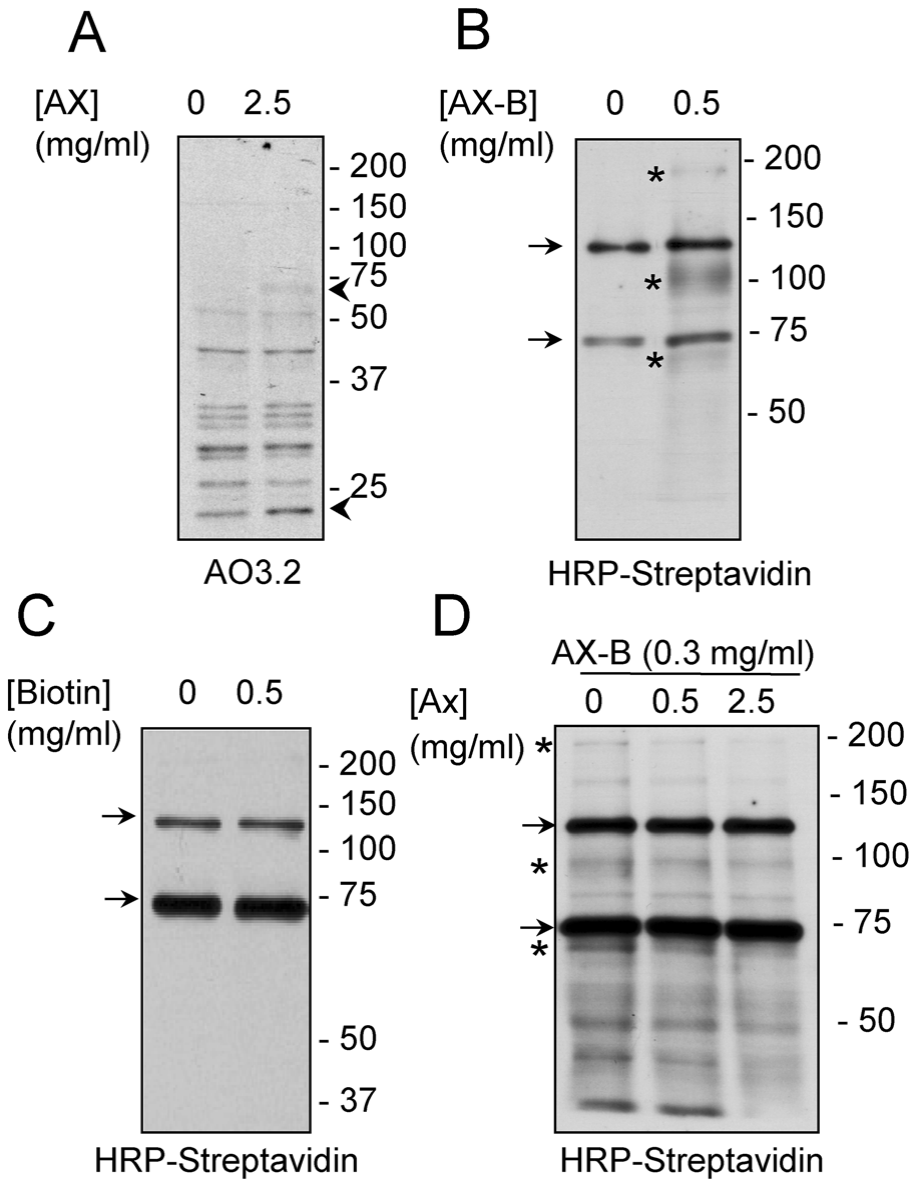
Detection of intracellular AX-B-protein adducts. (A) Raw264.7 murine macrophages were incubated in the absence or presence of AX for 24 h, after which, formation of AX-protein adducts was assessed by SDS-PAGE of cell lysates and detection with AO3.2 antibody. The position of two of the bands detected above background is marked by arrowheads. (B) Cells were incubated with the indicated concentrations of AX-B and protein adducts present in cell extracts were detected by SDS-PAGE, transfer and detection with HRP-streptavidin. The position of endogenous biotinylated proteins is marked by arrows and that of the major labeled proteins is indicated by asterisks. (C) Cells were incubated with biotin and the presence of biotinylated proteins was assessed as in (B). (D) Cells were incubated with the indicated concentrations of AX for 24 h, after which, AX-B was added at 0.3 mg/ml and incubation was continued for an additional 24 h. Incorporation of AX-B into proteins was assessed as above.

**Figure 9:**
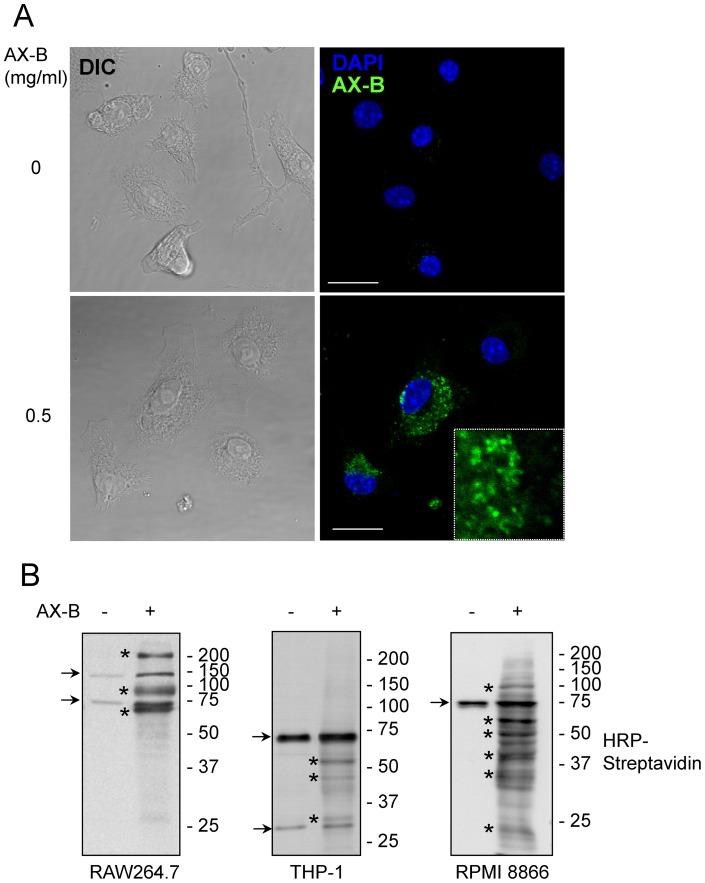
Localization and cell-type specificity of AX-B adducts. (A) RAW264.7 macrophages were cultured in the absence or presence of 0.5 mg/ml AX-B in serum-free medium for 24 h, after which, cells were fixed with paraformaldehyde and biotin positive structures were visualized by staining with Alexa-488-streptavidin. Nuclei were stained with DAPI. Shown is a single z section at mid cell height. DIC, differential interference contrast. Bar, 20 μM. (B) RAW264.7 cells (left panel), monocytic THP-1 (middle panel) and B lymphoma cells (right panel) were incubated with AX-B and the presence of AX-B-protein adducts was analyzed by SDS-PAGE and biotin detection with HRP-streptavidin and ECL. Typically, aliquots from total protein extracts containing 15 μg of protein were analyzed. Endogenous biotinylated polypeptides are marked by arrows and the position of the polypeptides showing higher biotin incorporation after AX-B treatment is indicated by asterisks.

According to the hapten hypothesis, drugs could either bind to extracellular proteins that would be endocytosed by cells, or bind to intracellular components. In both cases, drug-protein conjugates would be proteolysed and peptides presented on the cell surface [3]. Therefore, we attempted the detection of AX or AX-B-adducts in cells (Fig. 8). Incubation of RAW264.7 murine macrophages with high concentrations of AX followed by immunological detection revealed only a weak increase in the signal of certain bands over the background (Fig. 8A). Interestingly, incubation with AX-B resulted in the incorporation of the biotinylated antibiotic into several discrete bands, the most intense of which showed apparent molecular weights of 70, 100 and 200 kDa (Fig. 8B). These bands were distinct from the major endogenous biotinylated proteins, which appeared at 75 and 125 kDa and likely correspond to propionyl CoA and pyruvate carboxylases, respectively [32]. The increase in the biotin signal associated to the putative AX-B targets was not due to the extra supply of biotin since incubation of RAW264.7 cells with biotin did not result in biotin labeling of proteins (Fig. 8C). Interestingly, incubation of macrophages in the presence of an excess AX reduced the formation of adducts containing AX-B (Fig. 8D). In order to explore the localization of AX-B adducts we took advantage of the possibility of detecting biotin by confocal microscopy using fluorescent streptavidin (Fig. 9A). In these assays cells were incubated with AX-B in the absence of serum to minimize the potential contribution of serum proteins to the signals observed. Control cells showed a nearly undetectable perinuclear signal, typical of the mitochondrial localization of various endogenous biotinylated proteins [19]. In contrast, cells incubated in the presence of AX-B showed bright biotin-positive intracellular structures distributed throughout the cytoplasm. A blot of an experiment run in parallel, showing the main adducts detected under these conditions, is depicted in Fig. 9B (left panel). Next, we were interested in exploring whether the formation of AX-B protein adducts displayed cell type-specific features. As shown in Fig. 9B (middle and right panels), modified proteins in extracts from AX-B-treated THP-1 monocytic and B-lymphoma cells show different patterns, and differ also from those observed in RAW264.7 macrophages. These results indicate that the nature or the processing of the haptenated proteins may be cell type-dependent. Although at present the mechanism(s) of entry of AX-B or AX-B-protein conjugates into cells are not known, these observations indicate the presence of intracellular AX-B-protein adducts, thus raising the possibility that cellular proteins could contribute to the formation of haptens involved in the development of adverse reactions towards AX.

## Discussion

Drug binding to proteins plays a key role in the development of drug allergy. Here we have prepared and characterized a biotinylated derivative of AX (AX-B) and evaluated its ability to modify serum and/or cellular proteins, as indicated by 1D and 2D-electrophoresis analysis, as well as its recognition by anti-AX antibodies, to explore its potential as a tool for the study of protein haptenation. Our results show the usefulness of AX-B in the detection of adducts with serum proteins with high sensitivity, as well as in the detection of intracellular adducts. These properties can be exploited to obtain novel information on the mechanisms of the allergic reactions towards AX.

Biotinylated derivatives of reactive drugs or endogenous compounds have been widely used as tools to identify the targets of the parent compounds [19], [33], [34]. These approaches take advantage of the high affinity of the avidin-biotin interaction, characterized by an affinity constant of 10^15^ M^-1^, which in general appears to be three to six orders of magnitude higher than for the interaction of ligands with their specific antibodies [35], this property likely underlying the superior sensitivity of these methods. Here we have observed that both AX and AX-B display similar reactivity towards simple amines. Moreover, both compounds compete for their binding to proteins, suggesting that they may bind to common sites. In addition, we have observed that both compounds bind to the same serum targets, with the only difference of a weak binding to haptoglobin, which is undetectable using immunological AX detection, but is visible with AX-B due to the higher sensitivity of this method. Thus, both compounds seem to display similar binding to proteins. Nevertheless, it should be taken into account that the presence of the biotin moiety may impose steric impediments for binding to some targets or it may shield part of the molecule [36]. Indeed, AX-B adducts are not recognized by a monoclonal antibody directed towards the lateral chain of the AX molecule, which in AX-B is modified by the attachment of the biotin moiety. Also, results from RAST inhibition assays indicate that recognition of AX-B by the sera of non-selective patients is similar to that of AX, but it is poorer in the case of patients with allergy selective towards AX, whose IgE recognize AX lateral chain. Therefore, a detailed characterization of the binding of AX to the individual targets identified through the use of AX-B would be necessary. An interesting aspect of the RAST inhibition studies as well as of other *in vitro* tests using “free” β-lactams is that, during incubation, adducts of the β-lactams assayed and the proteins present in the assay, either from serum or from cells, will be formed (Ariza et al., unpublished observations). Therefore, these structures may also contribute to the selectivity observed and should be taken into account in the interpretation of the results.

Several strategies have been used to detect and identify targets and sites of action of β-lactam antibiotics, including various MS approaches recently developed, allowing a detailed characterization of HSA haptenation by various β-lactams [12], [37], [38]. Immunological approaches have also been used in various experimental systems. In particular, a selective modification of certain cytokines after *in vitro* incubation with high concentrations of BP, even in the presence of serum, has been evidenced by western blot [39]. In a recent work using also immunological detection, the only piperacillin adducted protein detected in the cell culture medium of T-lymphocytes incubated with this antibiotic was albumin. Moreover, no cellular adducts were detected [40]. Nevertheless, our results indicate that highly sensitive methods can allow the detection and identification of novel AX targets in serum and in cell culture, either in cells or in the extracellular medium. In fact, when the supernatant from cells cultured in the presence of AX-B was analyzed by blot and detection with streptavidin, a whole array of adducts was observed (results not shown). Moreover, we have clearly observed the presence of intracellular protein adducts by confocal fluorescence microscopy. Intracellularly detected protein adducts could correspond to extracellular proteins internalized by cells, either from the culture medium or from the plasma membrane, or to intracellular proteins. Interestingly, the covalent binding of AX to HLA class I molecules present at the membrane of B cells overexpressing these molecules has been reported [41]. However, under our conditions, the plasma membrane of AX-B-treated macrophages showed negligible biotin labeling. Interestingly, our results also indicate that AX-B may form different adducts in distinct cell types. Therefore, it would be interesting to assess whether binding of AX-B to membrane proteins can be observed in other cell types or under different culture conditions. On the other hand, the presence of intracellular targets for covalent modification by AX may increase the panoply of potential antigenic determinants generated by treatment with the antibiotic.

AX is known to enter mammalian cells and several transporters have been described that mediate the entry of β-lactam antibiotics, especially in kidney and liver cells [42], [43]. In addition, intracellular AX has been detected by immunocytochemistry, using an anti-AX antibody, in nuclei and cytoplasm of absorptive epithelial cells of the intestine and in hepatocytes [10]. Therefore, the nature of intracellular AX adducts and their potential role in allergy towards AX deserves future investigation.

From various studies, the concept of the selectivity of protein modification by AX and other β-lactams is beginning to emerge. The results herein presented also point towards a selective modification since some abundant serum proteins appear not to be modified by either AX or AX-B. Modification of HSA *in vitro* and *in vivo* by β-lactams shows selectivity, with some residues being preferentially modified. Moreover, in cell extracts, only some polypeptides show AX-B incorporation. Residue reactivity and conformational factors which favor β-lactam docking may play an important role in this selectivity [12], [40]. Identification and characterization of additional targets will help define the structural factors favoring AX addition.

In summary, our results show that use of highly sensitive approaches such as labeling with biotinylated analogs will allow the detection of novel targets of AX. Detailed structural information on the binding sites on various targets will provide potential new antigenic determinants to be used in diagnostic procedures and in studies on the mechanisms of AX-induced allergy.
